# Deep learning based prediction of necessity for orthognathic surgery of skeletal malocclusion using cephalogram in Korean individuals

**DOI:** 10.1186/s12903-021-01513-3

**Published:** 2021-03-18

**Authors:** WooSang Shin, Han-Gyeol Yeom, Ga Hyung Lee, Jong Pil Yun, Seung Hyun Jeong, Jong Hyun Lee, Hwi Kang Kim, Bong Chul Kim

**Affiliations:** 1 Safety System Research Group, Korea Institute of Industrial Technology (KITECH), Gyeongsan, Korea; 2 School of Electronics Engineering College of IT Engineering, Kyungpook National University, Daegu, Korea; 3 Department of Oral and Maxillofacial Radiology, Daejeon Dental Hospital, Wonkwang University College of Dentistry, Daejeon, Korea; 4 Department of Oral and Maxillofacial Surgery, Daejeon Dental Hospital, Wonkwang University College of Dentistry, Daejeon, Korea

**Keywords:** Cephalogram, Machine learning, Machine intelligence, Orthognathic surgery

## Abstract

**Background:**

Posteroanterior and lateral cephalogram have been widely used for evaluating the necessity of orthognathic surgery. The purpose of this study was to develop a deep learning network to automatically predict the need for orthodontic surgery using cephalogram.

**Methods:**

The cephalograms of 840 patients (Class ll: 244, Class lll: 447, Facial asymmetry: 149) complaining about dentofacial dysmorphosis and/or a malocclusion were included. Patients who did not require orthognathic surgery were classified as Group I (622 patients—Class ll: 221, Class lll: 312, Facial asymmetry: 89). Group II (218 patients—Class ll: 23, Class lll: 135, Facial asymmetry: 60) was set for cases requiring surgery. A dataset was extracted using random sampling and was composed of training, validation, and test sets. The ratio of the sets was 4:1:5. PyTorch was used as the framework for the experiment.

**Results:**

Subsequently, 394 out of a total of 413 test data were properly classified. The accuracy, sensitivity, and specificity were 0.954, 0.844, and 0.993, respectively.

**Conclusion:**

It was found that a convolutional neural network can determine the need for orthognathic surgery with relative accuracy when using cephalogram.

## Background

Using deep learning algorithms, artificial neural networks automatically extract the most characteristic features from data and return an answer [1, 2]. Deep learning has been introduced in various fields, and its usefulness has been proven [1–5]. Deep learning has demonstrated excellent performance in computer vision including object, facial and activity recognition, tracking and localization [6]. Image processing and pattern recognition procedures have become key factor in medical segmentation and diagnosis [7]. In particular, detection and classification of diabetic retinopathy, skin cancer, and pulmonary tuberculosis using deep learning-based convolutional neural network (CNN) models have already demonstrated very high accuracy and efficiency, with promising clinical applications [7]. In the dental field, this could perform the radiographic detection of dental pathology such as periodontal bone loss, dental caries, benign tumor and cystic lesions [7–10]. Deep learning was also applied to the diagnosis of dentofacial dysmorphosis using photographs of the subjects [11].

Patients presenting dentofacial deformities are commonly subject to combine orthodontic and surgical treatment[12]. A maxillofacial skeletal analysis is an important part of diagnosis and treatment planning [13], and it is used to assess the vertical, lateral, and anteroposterior positions of the jaws using posteroanterior (PA) and lateral (Lat) cephalogram. Accurate diagnosis has been based on proper landmark identification by cephalogram and it is essential to a successful treatment [13, 14]. The application of deep learning algorithms to cephalometric analysis has been studied, and many approaches have focused on the detection of cephalometric landmarks [15, 16]. However, there are numerous limitations to such methods; for example, the data on a detected landmark are significantly influenced by the skill of the observer, and the process is time-consuming [16]. Considering the high potential of errors and bias associated with conventional diagnostic methods through landmark detection, efforts have been made to eliminate the process of cephalometric landmark detection. A CNN-based deep learning system was proposed for skeletal classification without the need for landmark detection steps [17]. The network exhibited > 90% sensitivity, specificity, and accuracy for vertical and sagittal skeletal diagnosis.

However, no studies have been conducted to evaluate the need for orthognathic surgery using deep learning networks with cephalogram. In medical and dental fields, deep learning networks can be used for screening or provisional diagnosis rather than a final diagnosis and treatment plan. If a deep learning network could automatically evaluate patients whether orthognathic surgery is required, it can be applied as a useful screening tool in the dental field. In addition, since the image feature extraction process is included in the deep learning model, new information that is closely related to the necessity of orthognathic surgery can be identified.

## Methods

### Datasets

In this study, transverse and longitudinal cephalograms of 840 patients (Class ll: 244, Class lll: 447, Facial asymmetry: 149), who visited Daejeon Dental Hospital, Wonkwang University between January 2007 and December 2019 complaining about dentofacial dysmorphosis and/or a malocclusion, were used for the training and testing of a deep learning model (461 males and 379 females with a mean age of 23.2 years and an age range of 19–29 years, SD: 3.15). All of the patients were identified for necessity of orthognathic surgery to manage dentofacial dysmorphosis and malocclusion Class II and III. Adolescents with incomplete facial growth or those with a congenital deformity such as cleft lip and palate, infection, trauma, or tumor history were excluded. The cephalograms were obtained using a Planmeca Promax® (Planmeca OY, Helsinki, Finland), and the images were extracted in Dicom format. The original image had a pixel resolution of 2045 × 1816 with a size of 0.132 mm/pixel.

All radiographic images were annotated by two orthodontists, three maxillofacial surgeons, and one maxillofacial radiologist. Point A–nasion–point B (ANB) and a Wits appraisal were used for diagnosing the sagittal skeletal relationship. Jarabak’s ratio and Björk’s sum were used for determining the vertical skeletal relationship. One expert (B.C.K.) showed the analyzed images and propose the classification, and other experts discussed about the images and finish the labeling.

Based on the consensus of six specialists, patients who did not need orthognathic surgery were classified as Group I (622 patients—Class ll: 221, Class lll: 312, Facial asymmetry: 89). Group II (218 patients—Class ll: 23, Class lll: 135, Facial asymmetry: 60) was set up for the patients requiring orthognathic surgery owing to skeletal problems such as facial asymmetry, retrognathism, and prognathism. Although many factors other than the skeletal part (patient preference, patient soft tissue type, or operator preference) are considered to determine the final operation, this study evaluated only the need for surgery by skeletal factors obtained using cephalogram.

### Data composition and augmentation

The dataset was extracted by random sampling and was composed of training, validation, and test sets. The ratio of the sets was 4:1:5. The number of samples of the case requiring surgery consisted of 273, 30, and 304 for the train, validation, and test, respectively, and the number of samples of the case not requiring surgery consisted of 98, 11, and 109, respectively. Shift, contrast, and brightness variations were used as the augmentation method to help with the generalization. Factors regarding the amount of shift, increase (or decrease) in brightness, and increase (or decrease) in brightness contrast were randomly determined within a 10% range for each iteration.

### Learning details

The batch size was 16 images, and a stochastic gradient descent method was used to optimize the model. We trained the model at a learning rate of 0.1, momentum of 0.9, and weight decay coefficient of 0.0005. PyTorch was used as a framework for the experiment.

The hardware and software environment specifications are as follows.

- CPU: Intel i9-9900 k
- RAM: 64 GB
- GPU: NVIDIA Geforce RTX 2080 Ti
- Framework: Pytorch

### Network architecture

**Fig. 1:**
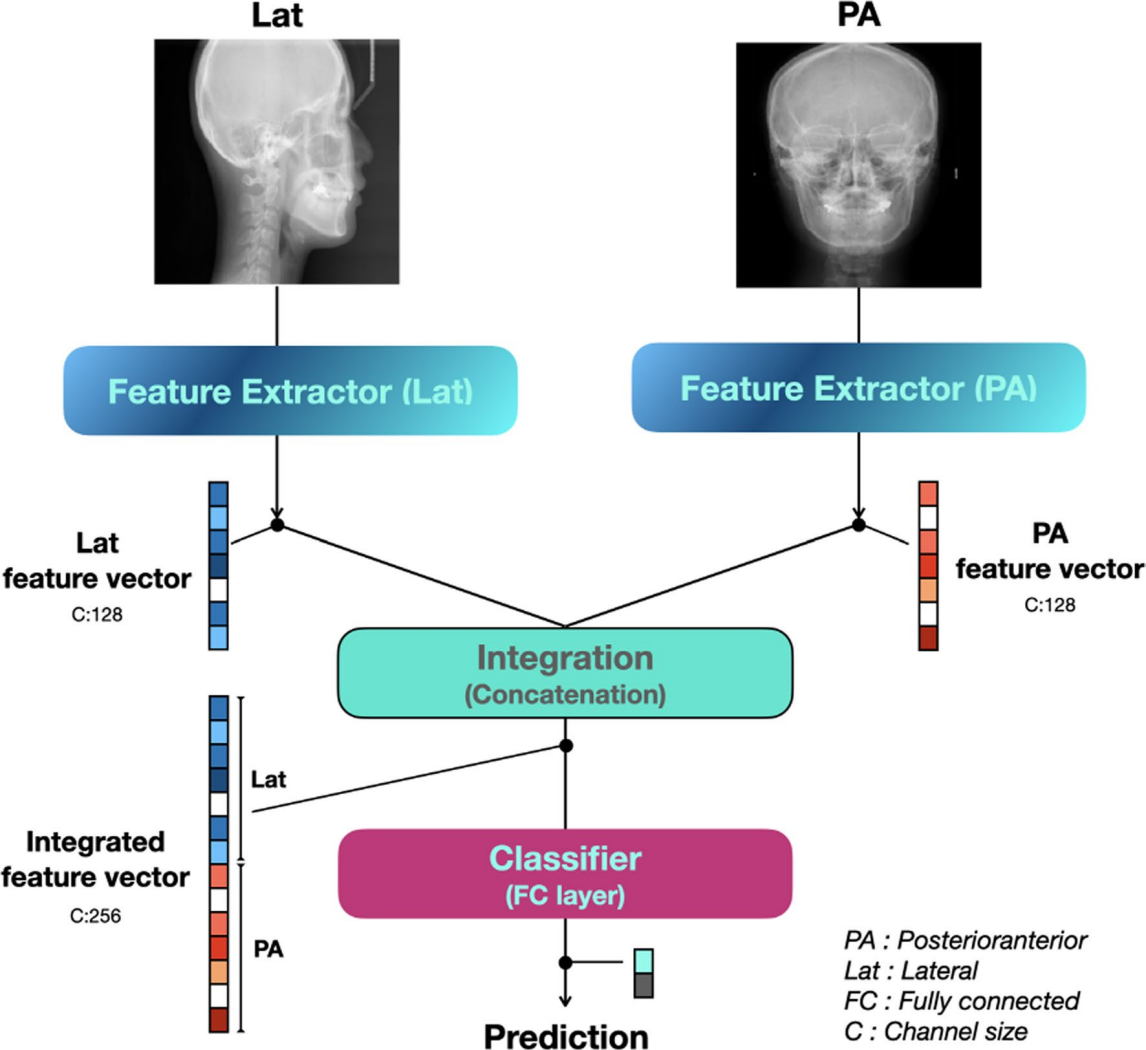
Structure of the entire model consisting of a feature extractor for feature extraction, concatenation part for merging features extracted from each side image, and classifier for classification based on the combined features. *PA* posteroanterior, *Lat* lateral, *FC* fully connected, *C* channel size

Figure 1 shows the structure of the entire model. It comprises a feature extractor for feature extraction, concatenation part for merging the features extracted from each side image, and classifier for classification based on the combined features.

**Fig. 2:**
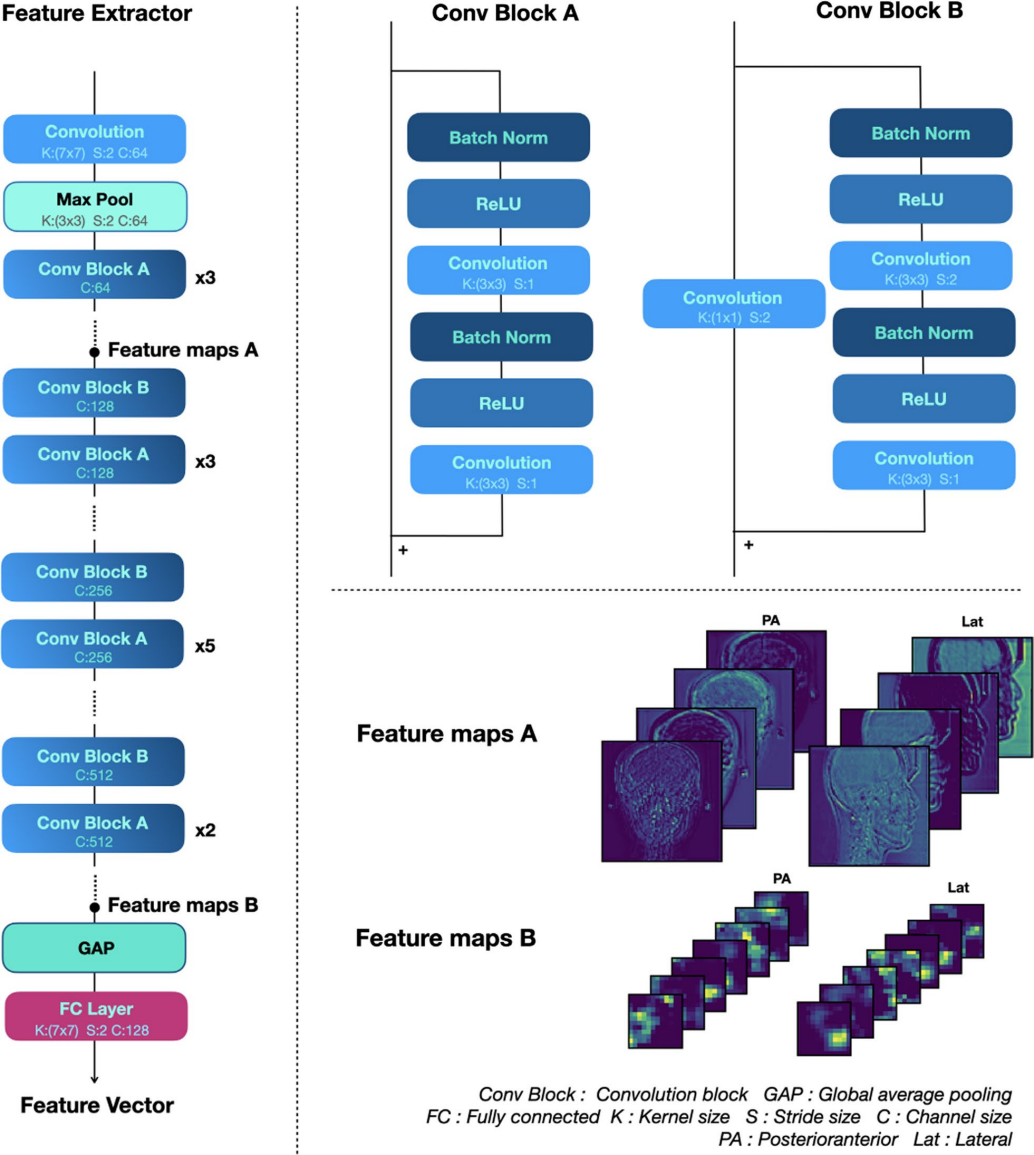
Components of the feature extractor in more detail. The backbone network of the feature extractor employs ResNet34, with convolution blocks stacked hierarchically. Utilizing pre-trained models with massively sized datasets such as ImageNet helps extract generalized features, even if it doesn't help extract task-specific features. Additionally, it helps improve the convergence speed of the training. Therefore, all parameters in the feature extractor except the fully connected layer are initialized to the parameters of the model pre-trained with ImageNet. The repetitive operations by the hierarchical architecture encode the features of the input image into an abstract feature vector. *Conv Block* convolution block, *GAP* global average pooling, *FC* fully connected, *K* kernel size, *S* stride size, *C* channel size, *PA* posteroanterior, *Lat* lateral

The proposed multi-side view independently extracts features from the feature extractor on each side and creates an integrated feature vector by concatenating the extracted feature vectors. Figure 2 shows the components of the feature extractor in more detail. The backbone network of the feature extractor employs ResNet34 [18], with convolution blocks stacked hierarchically. All weights in the feature extractor except the fully connected layer are initialized to the weight of the model pre-trained using ImageNet prior to training. The repetitive operations by the hierarchical architecture encode the features of the input image into an abstract feature vector.

Feature maps A and B in Fig. 2 show the output of the initial convolution block and the output of the last convolution block, demonstrating the abstraction process of features in the input image. The output feature maps of the initial convolution block retain the shape of the input image while the low-level features are activated. However, the feature maps of the last convolution block are compressed, and thus their shape cannot be recognized, although they activate high-dimensional areas such as the mandible and frontal bone.

The convolution block consists of two convolution layers, each of which contains a batch normalization operation and an activation function. The activation function uses a rectified linear unit. The convolution block of ResNet used as a backbone network has a skip connection that adds an input to the output. This structure allows the object of learning to be the residual function of an input and output. This mechanism is called residual learning. The residual block can minimize the lost information as it propagates from the input layer to the output layer. In addition, it can prevent the vanishing gradient problem of the gradient becoming considerably small during the back-propagation.

### Statistical analysis

A statistical analysis was performed by calculating the accuracy, sensitivity, and specificity, using the following equations, based on the confusion matrix shown in Table 1.

$$Accuracy = \frac{TP + TN}{{TP + TN + FN + FP}}$$

$$Sensitivity = \frac{TP}{{TP + FN}}$$

$$Specificity = \frac{TN}{{TN + FP}}$$

**Table 1:** Classification results

	Group I (Ground truth)	Group II (Ground truth)
Normal (Prediction)	302 (True Positive; TP)	17 (False Positive; FP)
Surgery (Prediction)	2 (False Negative; FN)	92 (True Negative; TN)

## Results

**Fig. 3:**
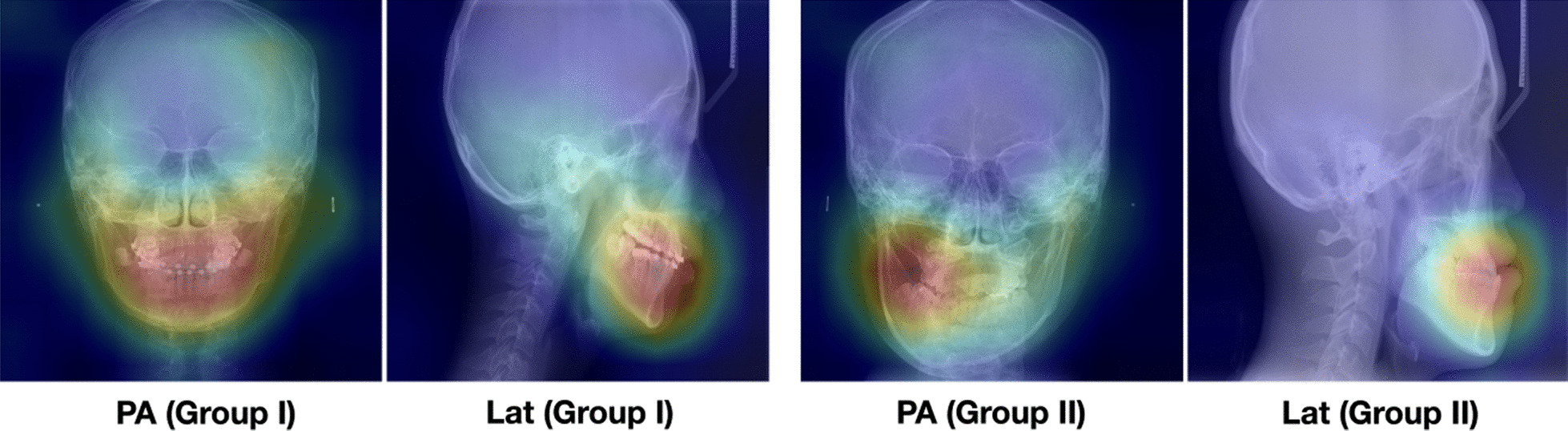
Feature map visualization. The teeth and maxillofacial area are highlighted. *PA* posteroanterior, *Lat* lateral

Table 1 shows the classification results in a confusion matrix, and 394 out of a total of 413 test data were properly classified. The accuracy, sensitivity, and specificity were 0.954, 0.844, and 0.993, respectively. The inference time we measured took about 64 ms. In addition, the visualized feature map of the feature extractor is overlapped with the original X-ray image (Fig. 3). The activation value is strong around the teeth and jawbone.

## Discussion

To achieve a more accurate judgment regarding the necessity of orthognathic surgery, the proposed model was designed to comprehensively consider the PA and Lat cephalogram together. Because two types of cephalograms were obtained from the same patient, both images had to be evaluated comprehensively for an accurate evaluation of a single patient. As the morphological features of each image (PA and Lat) were completely different, the features had to be extracted using an independent CNN model. The feature map of the last convolution layer of the feature extractor was converted into a vector through global average pooling (GAP). Feature information embedded as a vector by GAP can be considered together even if the image size is different. Flattening can also be used to convert the feature maps into vectors. However, if a sufficient number of features are already encoded channel-wise, a GAP is effective in preventing an overfitting and reducing the number of parameters [19].

Because the features extracted in the deep learning model are not engineered by humans but build automatically by training, interpretation is difficult. Therefore, it is difficult to tracing the evidence for the model's inference result. Feature visualization was applied to visualize those areas within an image that were particularly relevant for the decision of the network. Figure 3 shows the image overlapped with the input image after averaging channel-wise the last layer feature maps of the feature extractor. Regions of high and low influence are color-coded to visualize the relevant areas for the decision of the model. As a meaningful result, the teeth and maxillofacial area are highlighted in the visualization map. Similar to an orthodontist, a maxillofacial surgeon, or a maxillofacial radiologist, the deep learning network also focused on these sites and evaluate the need for orthognathic surgery.

The significance of this paper is that it simplifies the conventional cephalometric analysis process that selects and analyzes landmarks arbitrarily determined by humans from radiologic information consisting of countless patient factors. In addition, since the image feature extraction process is included in the deep learning model, new information closely related to the necessity of orthognathic surgery could be identified.

We added a part that fuses the features extracted from the Lat and PA images by each feature extractor in the proposed model. The results of this study indicate that, unlike previous studies that used only Lat cephalograms [17], PA and Lat cephalograms were used comprehensively and inferred. Since the patient's asymmetry must be evaluated as an important determinant of surgery, the simultaneous judgment of PA cephalogram has great clinical significance.

The proposed model is not for determine a specific surgery plan. Rather it serves as a general guide for deciding the necessity of orthognathic surgery for both patient and general practitioner of dentist. Since the deep learning-based prediction does not take much time, it can save time and cost of the process which lowers the entry barrier so patients can get a more appropriate diagnostic process. Also, it would have a great impact to optimize treatment time.

The limitation is that this study involves only Korean patients from only one hospital. Also, the number of cases was small. Further study should be accompanied to achieve public confidence.

## Conclusions

In this study, we exploited a deep learning framework to automatically predict the need for orthognathic surgery based on the cephalogram of the patients. A dataset of 840 case images was built and used to evaluate the proposed network. The accuracy, sensitivity, and specificity were 0.954, 0.844, and 0.993, respectively. We hope these results will facilitate future research on this subject and help general dentists screen patients complaining of dentofacial dysmorphosis and/or a malocclusion and propose an overall treatment plan. It will benefit not only oral and maxillofacial surgeon and orthodontist, but also general dentist. Deep learning program enables the process of standardized decision and help them understand the necessity of orthognathic surgery of the patient.

## Data Availability

The datasets generated and analyzed during the current research are not publicly available, because the Institutional Review Board of Daejeon Dental Hospital, Wonkwang University did not allow it, but they are available from the corresponding author on reasonable request.
